# Microstructure and optical properties of Ag/ITO/Ag multilayer films

**DOI:** 10.1186/1556-276X-8-424

**Published:** 2013-10-17

**Authors:** Zhaoqi Sun, Maocui Zhang, Qiping Xia, Gang He, Xueping Song

**Affiliations:** 1 School of Physics & Materials Science, Anhui University, Hefei 230601, People’s Republic of China

**Keywords:** Ag/ITO/Ag multilayer films, Microstructure, Optical properties

## Abstract

Transflective and highly conductive Ag/ITO/Ag multilayer films were prepared by magnetron sputtering on glass substrates. The microstructure and optical properties of Ag/ITO/Ag multilayer films were systematically investigated by X-ray diffraction, scanning electron microscopy, and ultraviolet-visible spectroscopy. The optical properties of the multilayer films were significantly influenced by the thickness of the Ag surface layer from 3.0 to 12.6 nm. The multilayer film of Ag9.3nm/ITO142nm/Ag9.3nm shows the best comprehensive property. It could satisfy the requirement for transflective LCD.

## Background

Indium tin oxide (ITO) thin films have low electrical resistance and high transmittance in the visible spectrum. Because of their unique photoelectrical properties, they play an important role in optoelectronic devices, such as flat displays, thin-film transistors, solar cells, and so on [1-6].

It is well known that transmissive LCD has low contrast ratio in bright light and high power consumption. Reflective LCD has low contrast ratio in weak light, and most of them belong to monochromatic LCD. However, transflective LCD possesses high contrast ratio in bright and weak light as well as low power consumption.

Ag is a noble metal with excellent photoelectrical properties. In addition to good conductivity, it has high reflectivity in the visible range and good chemical stability. Thus, Ag/ITO composite material is the optimizing material to make new transflective LCD. Miedziński reported the electrical properties of Ag/ITO composite films [7]. Choi fabricated ITO/Ag/ITO multilayer films and obtained a high-quality transparent electrode which has a resistance as low as 4 *Ω*/*ϒ* and a high optical transmittance of 90% at 550 nm [8]. Bertran prepared Ag/ITO films with a high transmittance (near 80%) in the visible range by RF sputtering and studied their application as transparent electrodes in large-area electrochromic devices [9]. Guillén prepared ITO/Ag/ITO multilayer films with visible transmittance above 90% by sputtering at room temperature and investigated the optical and electrical characteristics of single-layer and multilayer structures. Besides, the transmittance is found to be mainly dependent on the thickness of Ag film [10]. Although much work has paid more attention on the investigation of Ag/ITO/Ag multilayer films, few studies have been carried out to study their photoelectrical properties.

In this study, Ag/ITO/Ag multilayer films with various surface layer thicknesses have been prepared on a glass substrate by direct current (DC) magnetron sputtering. The microstructure and optoelectronic properties of the Ag/ITO/Ag films were investigated using X-ray diffraction (XRD), scanning electron microscopy (SEM), and ultraviolet-visible spectroscopy (UV-vis).

## Methods

The multilayer films were prepared by an ultrahigh vacuum multifunctional magnetron sputtering equipment (JGP560I, SKY Technology Development Co., Ltd, Shenyang, China). The multilayer films with a sandwich structure were deposited on glass substrates. The Ag layers were deposited by DC magnetron sputtering with a power density of 1.73 W/cm^2^, while the ITO coatings were deposited by radio frequency magnetron sputtering with a power density of 2.12 W/cm^2^. Ceramic ITO targets of In_2_O_3_:SnO_2_ disk (90:10 wt.%, 4N) and an Ag metal target (4N) were used for ITO and Ag layer deposition separately. The target-to-substrate distance was 60 mm. The base vacuum was 6.0×10^-4^ Pa, and the deposition pressure was 1.0 Pa with an argon (4N) flow rate of 45 sccm. During the deposition, the substrates were kept in room temperature.

The thicknesses of Ag/ITO/Ag multilayer films were controlled by sputtering time. Ag films and ITO films were firstly prepared respectively at a fixed time, and their thicknesses were tested using a surface profiler (XP-1, Ambios Technology, Santa Cruz, CA, USA). The deposition rate of Ag films and ITO films was calculated according to the sputtering time and film thicknesses. Then Ag/ITO/Ag multilayer films could be prepared with different sputtering time.

The multilayer films consist of three layers. The thicknesses of Ag surface layer vary from 3.0 to 12.6 nm while the Ag bottom layer keeps the same thickness of 9.3 nm. All samples have an ITO interlayer of 142 nm. Table 1 shows the thickness of the multilayer films.

**Table 1:** Microstructure parameters and the average reflectance with 300 to 900 nm of Ag/ITO/Ag multilayer films

**Samples**	**Compositions**	**Ag(111) grain size (nm)**	**Average reflectance *R *** **(%)**
Ag1/ITO/Ag	Ag 3.0 nm/ITO 142 nm/Ag 9.3 nm	2.477	22.04
Ag2/ITO/Ag	Ag 6.4 nm/ITO 142 nm/Ag 9.3 nm	5.955	25.07
Ag3/ITO/Ag	Ag 9.3 nm/ITO 142 nm/Ag 9.3 nm	11.945	28.98
Ag4/ITO/Ag	Ag 12.6 nm/ITO 142 nm/Ag 9.3 nm	19.885	31.12
ITO	-	-	23.76

The microstructure was analyzed with a MXP 18AHF X-ray diffractometer (MAC Science, Yokohama, Japan). The X-ray source was CuKα, with an accelerating voltage of 40 kV, a current of 100 mA, scanning range from 20° to 80°, glancing angle of 2°, scanning step of 0.02°, and scanning speed of 8°/min. The surface morphology of the films was studied with a Hitachi S-4800 type SEM (Hitachi, Tokyo, Japan). The optical properties were tested with a Shimadzu UV-2550 type ultraviolet-visible spectroscope (Shimadzu, Kyoto, Japan). The scanning range was from 300 to 900 nm, scanning step was 1 nm, and slit width was 2 nm.

## Results and discussion

### Microstructure analysis

**Figure 1:**
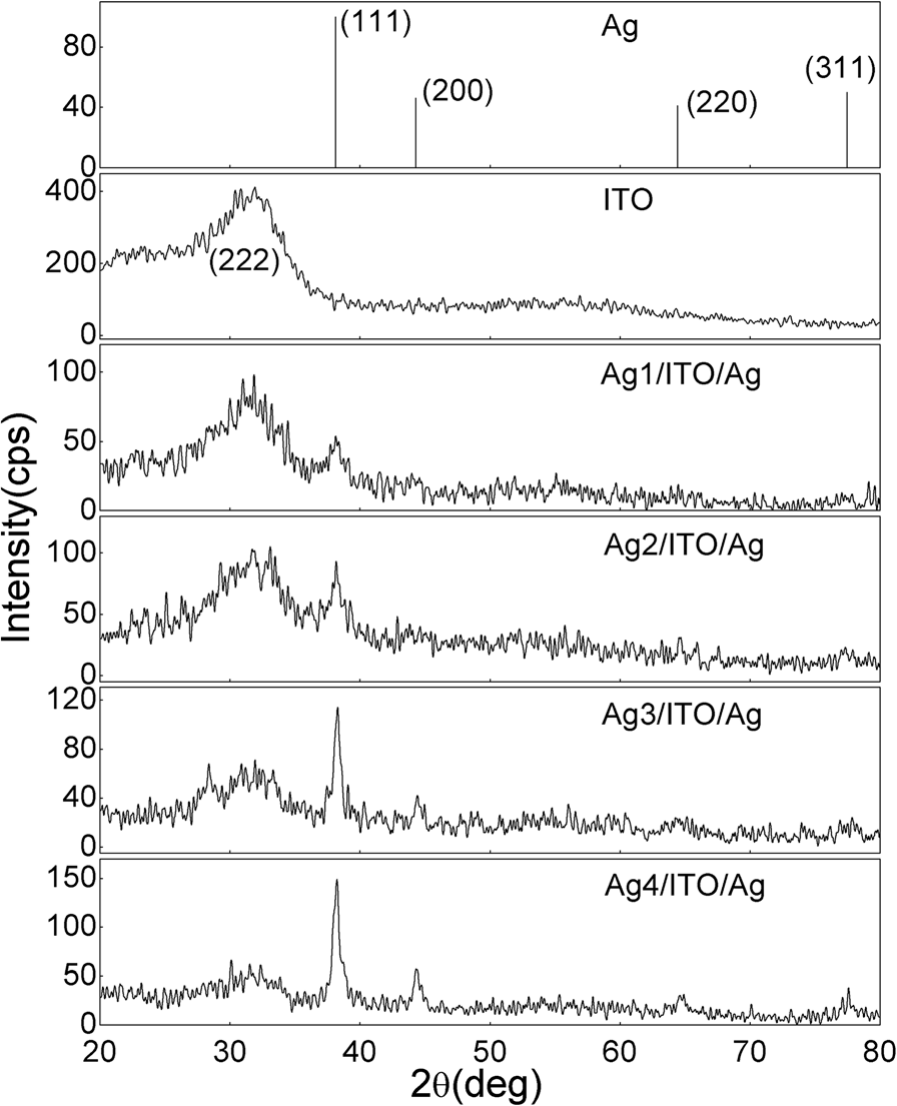
XRD patterns of the Ag, ITO, and Ag/ITO/Ag multilayer films.

Figure 1 shows the XRD patterns of the Ag, ITO, and Ag/ITO/Ag films. Based on Figure 1, it can be noted that broad In_2_O_3_ (222) diffraction peaks have been observed in the ITO and Ag/ITO/Ag films. As the thickness of the Ag surface layer increased, the Ag (111) preferred orientation intensified [11,12].

From Scherrer’s formula,

(1) D=Kλ/βcosθ

where *K* is 0.9, *λ* is 1.54056 Å, *β* is the full width at half maximum of the diffraction peak, and *θ* is the diffraction angle. The value of Ag grain size D(111) was calculated, and the results were listed in Table 1. With the increase of Ag surface layer thickness from 3.0 to 12.6 nm, the Ag grain size of all films increases.

**Figure 2:**
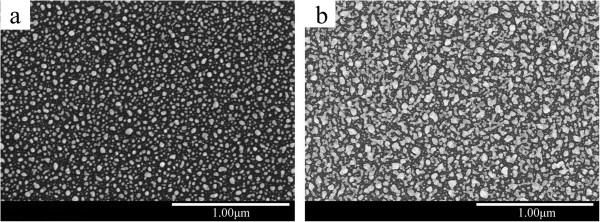
SEM micrographs of Ag films with different thicknesses: (a) 1.2 nm and (b) 1.8 nm.

Figure 2 shows the SEM micrographs of single-layer Ag films. According to Figure 2, it has been found that the surface morphology of the Ag film is critically dependent on its thickness. As shown in Figure 2a, the Ag nanoparticles are uniformly distributed in substrate. The Ag film forms in stable nuclei stage. As the thickness of the Ag film increases, randomly connected Ag islands appear, as shown in Figure 2b.

**Figure 3:**
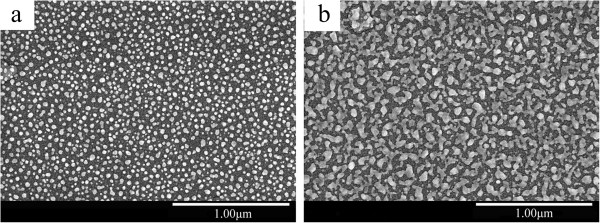
SEM micrographs of Ag/ITO/Ag multilayer films: (a) Ag2/ITO/Ag and (b) Ag3/ITO/Ag.

Figure 3 shows the SEM micrographs of Ag2/ITO/Ag and Ag3/ITO/Ag multilayer films. As shown in Figure 3a, the Ag nanoparticles are spherical and uniformly distributed in ITO films. The size of Ag nanoparticle is 5 to 60 nm. With increasing thickness of the Ag surface layer, randomly connected Ag network also appears, as shown in Figure 3b.

**Figure 4:**
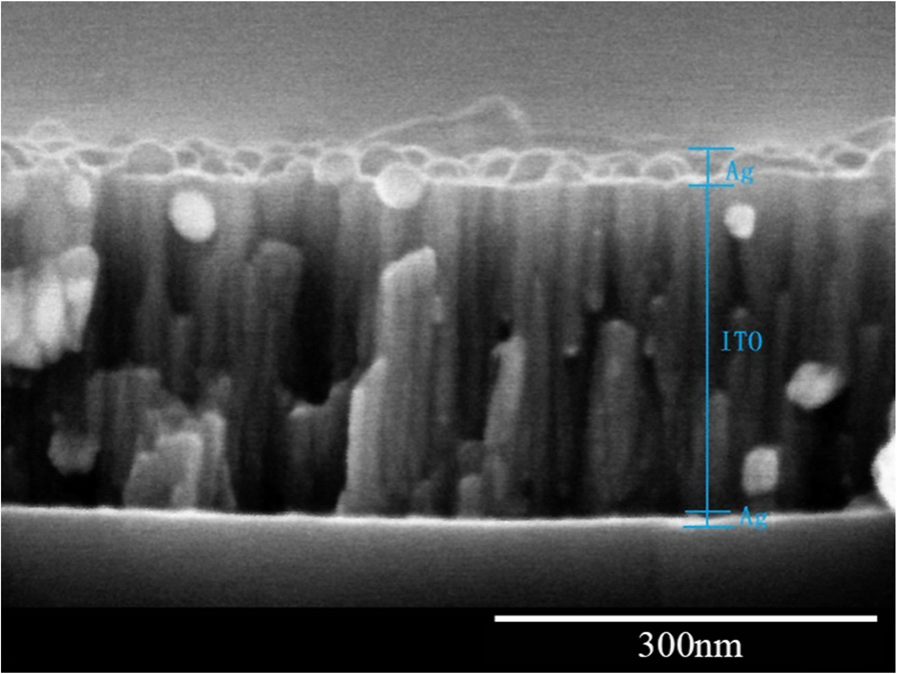
Cross-sectional SEM micrograph of Ag3/ITO/Ag multilayer film.

Figure 4 shows a cross-sectional SEM micrograph of Ag3/ITO/Ag multilayer film. The Ag surface layer, ITO interlayer, and Ag bottom layer forming the sandwich structure multilayer film have been observed clearly. From Figure 4, it has been seen that the Ag surface layer and bottom layer have a spherical cluster structure, and the interlayer of ITO film has a columnar structure.

### Optical properties

**Figure 5:**
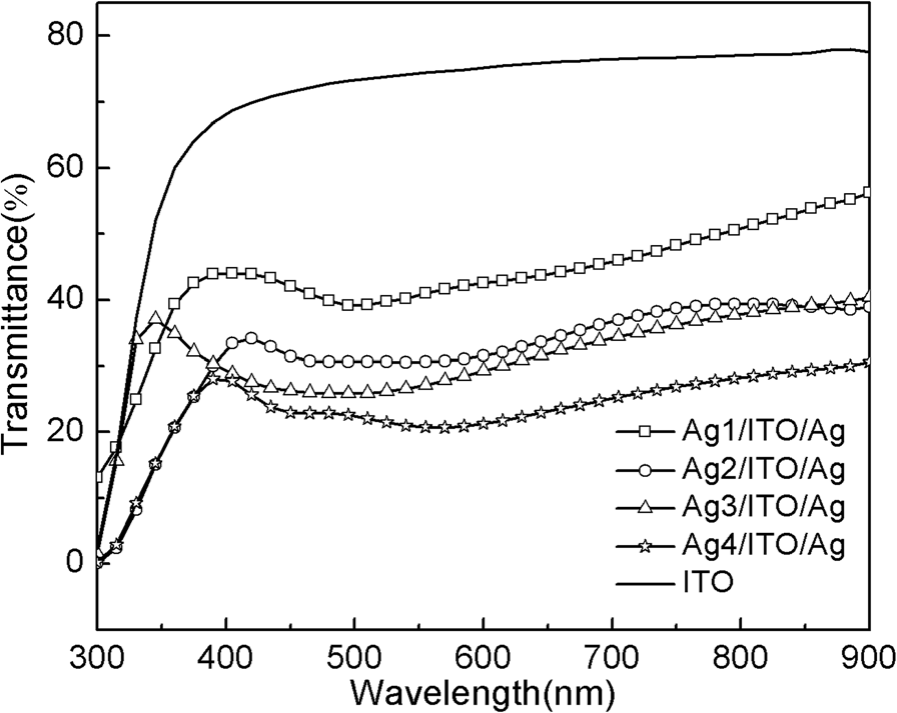
Transmittance spectra of Ag/ITO/Ag multilayer films.

Figure 5 shows the thickness-dependent transmittance spectra of the multilayer films changing wavelength from 300 to 900 nm. Compared with the bare ITO, the sandwich structure films have lower optical transmittance. It is suggested that the island structure of the thin Ag surface layer makes its transmittance low due to the large islands and the defects scattering incident light [9,13]. With the increase of Ag surface layer thickness from 3.0 to 12.6 nm, the transmittance of the multilayer films decreases, which is caused by the changes of the Ag surface layer first from a stable nuclei stage to randomly connected Ag island stage then to Ag network stage. Besides, Ag1/ITO/Ag, Ag2/ITO/Ag, and Ag3/ITO/Ag have low optical transmittance at about 500 nm. Ag4/ITO/Ag has low optical transmittance at about 450 and 550 nm. It is due to the surface plasmon resonance characterization of Ag.

**Figure 6:**
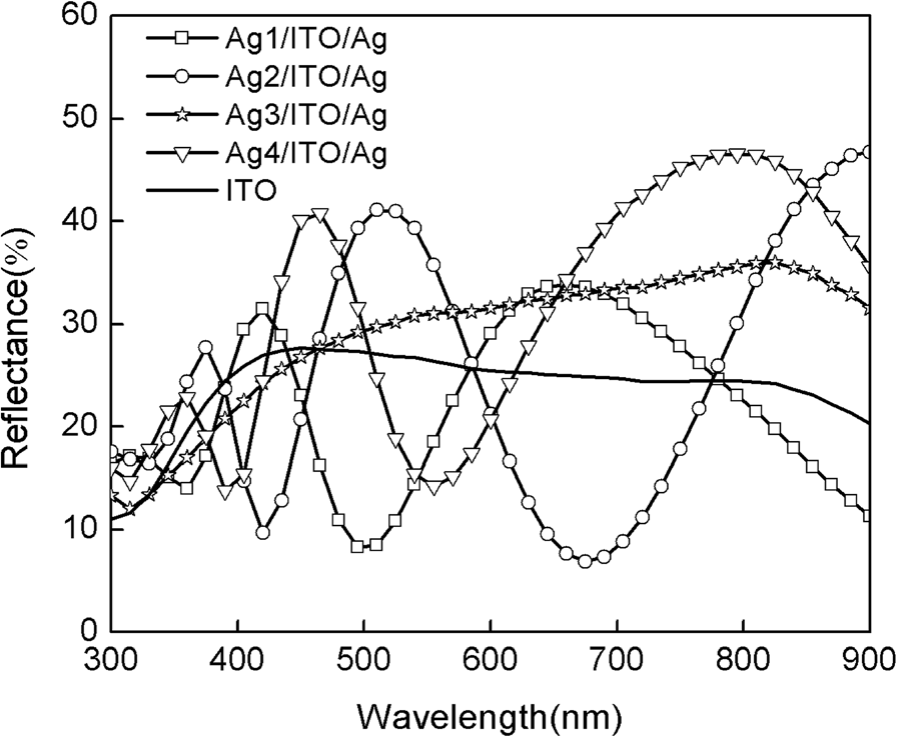
Reflectance spectra of the ITO and Ag/ITO/Ag multilayer films.

Figure 6 shows the reflectance spectra of the ITO and multilayer films. Based on Figure 6, it can be observed that multilayer Ag/ITO/Ag films show higher reflectivity than pure ITO film due to the high reflectivity of Ag. Table 1 calculated the average reflectance of the bare ITO and multilayer films. When the thickness of the Ag surface layer increases from 3.0 to 12.6 nm, the microstructure and surface morphology of the Ag surface layer changes a lot; the decrease of holes and defects in the films reduces the energy loss of light and the absorption of multilayer film, so the average reflectance of multilayer films increases from 22.04% to 31.12%. Besides, there is an interference phenomenon in the reflectance spectra of Ag1/ITO/Ag, Ag2/ITO/Ag, and Ag4/ITO/Ag; this will lead to uneven reflection and affect the quality of the LCD. The reflectance spectra of Ag3/ITO/Ag are relatively flat and can eliminate the influence of the interference phenomenon.

**Figure 7:**
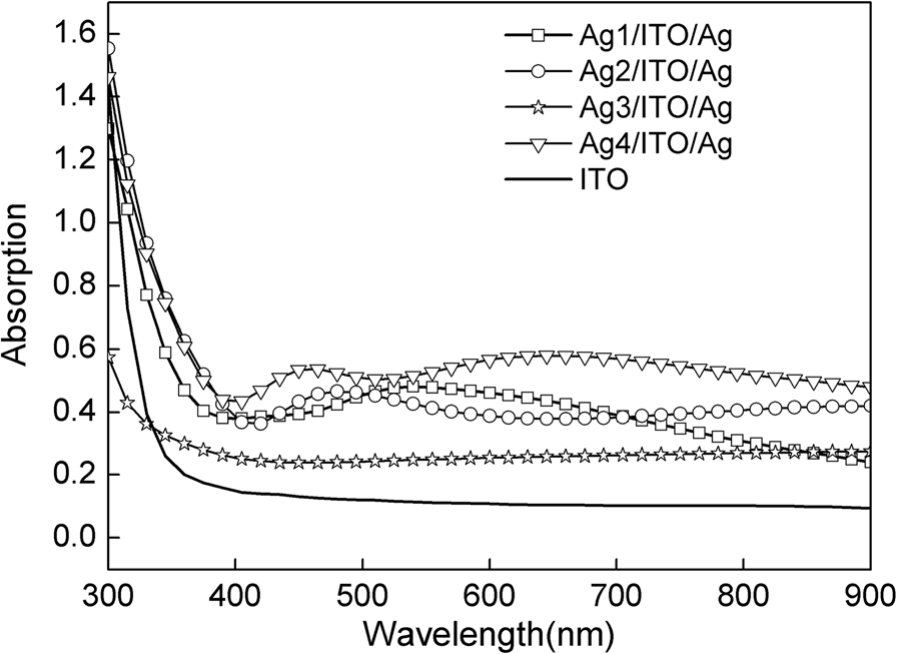
Optical absorption spectra of the ITO and Ag/ITO/Ag multilayer films.

Figure 7 shows the absorption spectra of the ITO and multilayer films. With increasing thickness of the Ag surface layer, the average transmittance of the multilayer films first increases then decreases. Compared with the bare ITO films, the absorption of multilayer films increases due to the introduction of a double Ag layer. However, the absorption of Ag1/ITO/Ag film is close to that of the bare ITO film, and no absorption peaks appeared.

## Conclusions

Ag/ITO/Ag multilayer films with different thicknesses of the surface Ag layer were prepared by magnetron sputtering on a glass substrate. Microstructural analysis shows that the multilayer films have a polycrystalline structure. As the thickness of the Ag surface layer increases, the preferred orientation of Ag (111) intensified. With increasing thickness of Ag surface layer, the transmittance spectra and reflectance spectra of Ag/ITO/Ag multilayer films decrease and increase, respectively. Ag3/ITO/Ag multilayer film shows the best comprehensive property and can be used as a potential transflective candidate in future LCD.

## Competing interests

The authors declare that they have no competing interests.

## Authors’ contributions

ZQS and QPX prepared the films and tested the surface topography. X-ray diffraction was investigated by XPS and MCZ. The optical properties were measured by GH. The calculations were carried out by ZQS who also wrote the manuscript. Besides, MCZ helped draft the manuscript. All authors read and approved the final manuscript.
